# An overview of the production and use of *Bacillus thuringiensis* toxin

**DOI:** 10.1515/biol-2022-0902

**Published:** 2024-08-06

**Authors:** Kaixiao Li, Mingzhu Chen, Jingyi Shi, Tian Mao

**Affiliations:** College of Life Science and Technology, Xinjiang University, 666 Shengli Road, Xinjiang Uygur Autonomous Region, Urumqi, 830000, People’s Republic of China; College of Textiles and Clothing, Xinjiang University, Xinjiang Uygur Autonomous Region, Urumqi, 830000, China; Graduate School of Xinjiang Medical University, Xinjiang Medical University, Xinjiang Uygur Autonomous Region, Urumqi, 830000, China

**Keywords:** *Bacillus thuringiensis*, research and application, probiotics, insecticidal mechanism

## Abstract

The widespread utilization of traditional chemical pesticides has given rise to numerous negative impacts, leading to a surge in interest in exploring environmentally friendly alternatives. *Bacillus thuringiensis* (Bt), a bacterium renowned for its insecticidal properties, produces *Cry* proteins during its lifecycle. These proteins have distinct advantages over traditional chemical pesticides, including higher environmental safety, broader insecticidal spectra, and lower pesticide residues. Consequently, the discovery and application of Bt hold immense significance in plant disease and pest management, as well as in plant protection. Currently, Bt preparations occupy a prominent position as the world’s largest and most widely used biopesticides. This article comprehensively reviews the fundamental aspects, insecticidal mechanisms, practical applications, and fermentation technologies related to Bt.

## 1 Introduction

*Bacillus thuringiensis* (Bt) is a Gram-positive bacterium known for its ability to produce spores and parasporal crystals. In 1901, the Japanese scientist Shigetane Ishiwatari isolated this rod-shaped bacterium from the larvae of *Bombyx mori* [1]. A decade later, in 1915, Berliner isolated the bacterium from the Mediterranean flour moth, an insect that had infested a flour mill in Thüringen, Germany, hence the official name *Bacillus thuringiensis* [2].

The earliest commercial production began in France in 1938 under the name Sporeine [3]. Subsequently, Hannay confirmed that these crystals were, in fact, toxic protein crystals responsible for the mass mortality of the Mediterranean flour moth [4]. By the early 1980s, Gonzalez et al. [5] revealed that the genes coding for crystal proteins were localized on transmissible plasmids. Over the past century, the classification of Bt has become increasingly clear and precise. Researchers have conducted extensive analysis on its structure, effective components, and insecticidal mechanisms, laying a solid foundation for its extensive application in agriculture, forestry, and pest control [6].

Today, Bt preparations are globally recognized as one of the most widely used biopesticides. Their environmental safety, broad insecticidal spectrum, and minimal pesticide residues make them an attractive alternative to traditional chemical pesticides.

## 2 Overview of Bt

### 2.1 Morphological characteristics of Bt

Bt is widely distributed in nature. Currently, tens of thousands of Bt strains have been isolated and preserved globally, originating from various sources such as soil [7], animals [8], plant surfaces [9], and feces [10]. The vegetative cells of Bt exhibit a rod-shaped morphology with blunt ends. When cultivated for a certain period, the vegetative cells transform into thicker sporangiums, which are larger than the vegetative cells. Subsequently, the sporangium ruptures, releasing oval-shaped spores along with parasporal crystal proteins. These crystal proteins can have various shapes, including obtuse rhombus, square, ellipsoid, and irregular forms.

### 2.2 Taxonomic status of Bt

In the ninth edition of Bergey’s Manual of Determinative Bacteriology, Bt is listed as one of the species in group 18 of the second category, with the characteristic of forming one or more accessory spore crystal proteins, while forming spores allows it to be distinguished from other *Bacillus strains*. Bt, *Bacillus cereus* (Bc), and *Bacillus anthracis* are closely related and belong to the Bc group. Despite the high similarity in their genome sequences, there are significant differences in the composition of their spores, which affect their survival abilities [11]. There is a particularly close evolutionary relationship between Bt and Bc, to the extent that 16S rDNA sequences alone cannot distinguish between the two species. However, more advanced techniques such as average nucleotide identity analysis and ribosomal multilocus sequence typing can successfully separate Bc and Bt into two distinct branches on the evolutionary tree [12]. This demonstrates the complexity and subtle differences within the *Bacillus* strains group, requiring sophisticated molecular techniques for accurate classification and identification.

## 3 Physicochemical properties of Bt

### 3.1 Insecticidal crystal proteins (ICPs)

**Figure 1:**
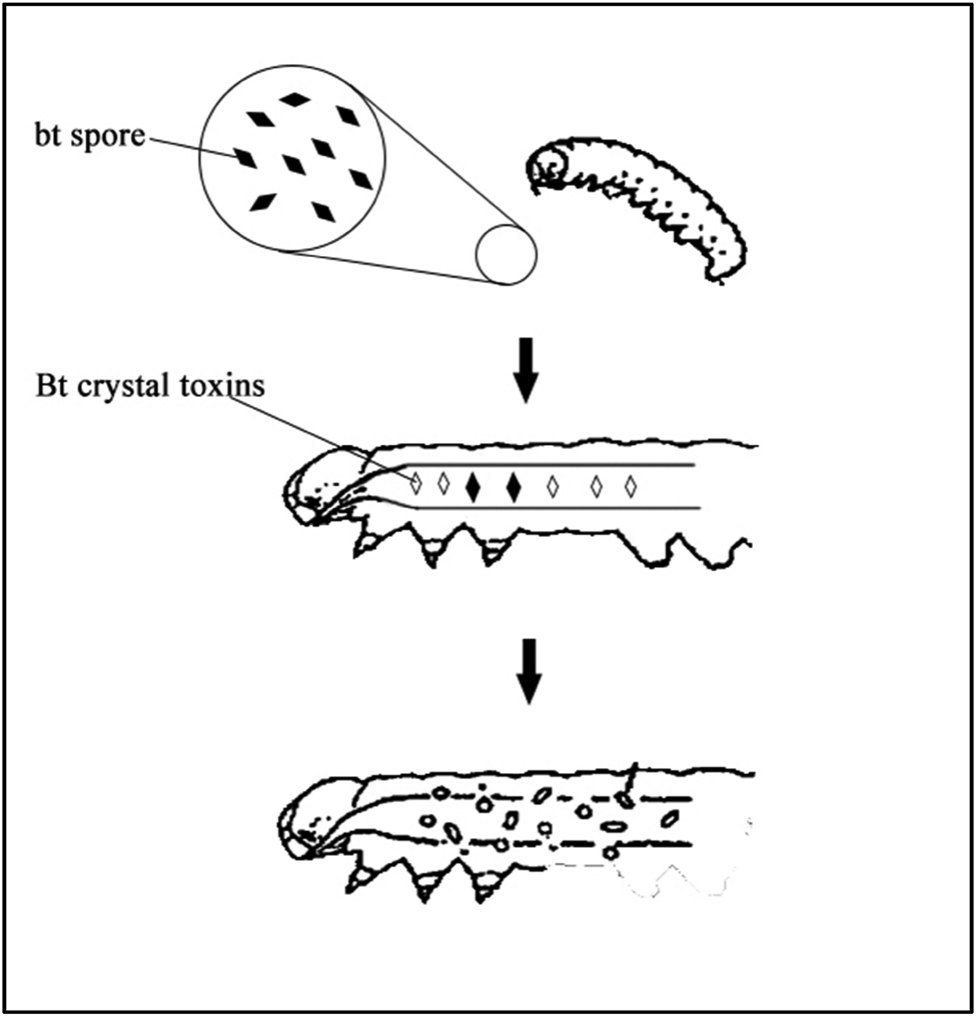
Insecticidal mechanism of parasporal crystals.

ICPs, also known as *δ*-endotoxins, produced by Bt are environmentally friendly active proteins. These proteins remain inactive in the acidic environment of the vertebrates’ gastrointestinal tract but become lethal to insects under the alkaline conditions found in their midguts [13]. Extensive research has revealed that the insecticidal process of ICPs follows a specific sequence of events. First, the parasporal crystals are lysed within the insect’s body. This lysis is followed by enzymatic activation, where the disulfide bonds within the ICPs are cleaved under alkaline conditions, releasing the protoxin. This protoxin is then further activated by trypsin enzymes, transforming it into an active toxin protein. Subsequently, the activated toxin protein binds to receptors on the midgut epithelial cell membrane. The toxic component of the protein, alpha-helix, inserts into the cytoplasmic membrane, creating holes or foci. This disrupts the membrane potential and leads to imbalances in cell permeability. As a result, the midgut undergoes necrosis, and there is damage to the peritoneal membrane and midgut epithelium. Finally, alkaline substances from the midgut enter the insect’s blood cavity, causing paralysis and eventually death [14]. The insecticidal mechanism of Bt is depicted in Figure 1.

### 3.2 Insecticidal characteristics and existing problems of Bt

Bt, as a microbial pesticide, has been widely used due to its unique insecticidal properties since its discovery. Overall, Bt preparations are considered safe and reliable as they can specifically target and eliminate harmful insects without harming animals and plants. In addition, it reduces the usage rate of chemical pesticides, and it is in line with the strategy of sustainable development of agricultural economy for it is harmless to the environment. Nevertheless, the widespread application of Bt inevitably poses some potential safety concerns. Certain Bt strains produce thuringiensin, also known as β-exotoxin, during their growth process. This exotoxin, secreted outside the cells, exhibits a broad insecticidal spectrum but is toxic to mammals, posing a potential safety hazard in agricultural applications [15].

Repeated application of insecticides to a specific insect population results in the survival of unaffected individuals who subsequently transmit their genetic traits to the subsequent generation. Over time, an increasing number of insect populations develop resistance to these insecticides. Unfortunately, insects have developed resistance to most widely used synthetic chemical insecticides. In 1979, the United Nations Environment Programme declared insecticide resistance as “one of the most serious environmental problems in the world.”

**Figure 2:**
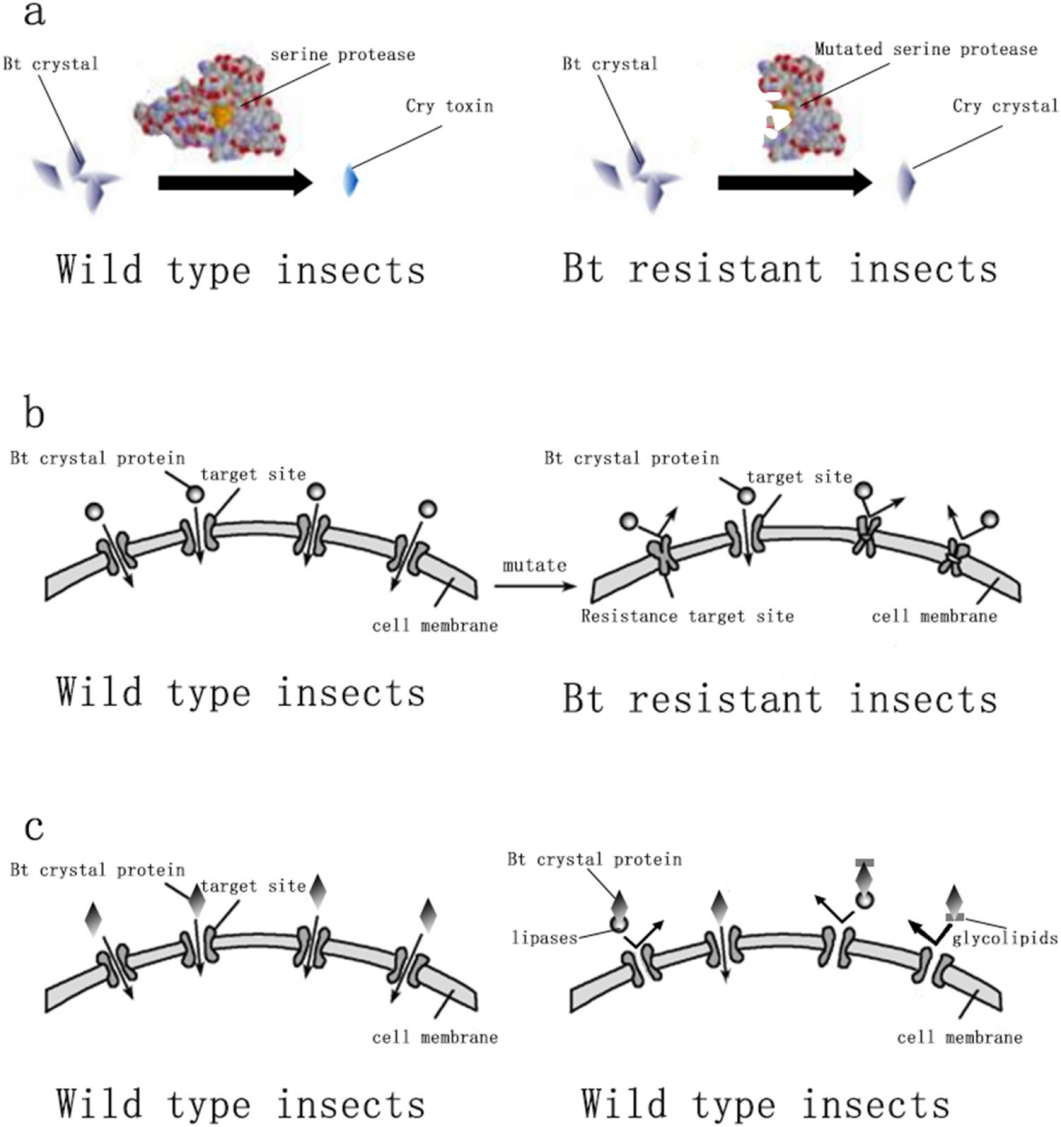
Bt resistance mechanism of insects. (a) Alteration in the activation process of Bt toxin. (b) Reduction or alteration in the binding of Bt toxin to midgut receptors. (c) Chelation of the Bt toxin with glycolipids and lipases.

Research studies indicate that the mechanisms of Bt resistance primarily arise from four aspects: alteration in the activation process of Bt toxin [16], chelation of Bt toxin with glycolipids and lipases, enhancement of the immune mechanisms of pests [17], and reduction or alteration in the binding of Bt toxin to midgut receptors [18]. The resistance mechanism of insects to Bt toxin is shown in Figure 2. Bt resistance can lead to poor crop quality, increased pesticide production costs, and the emergence of secondary pests due to the large quantities of pesticides used to eliminate the target pest.

In conclusion, despite the significant insecticidal effect and environmental friendliness of Bt, its application must take into account safety concerns, particularly the emergence of insect resistance. Therefore, continuous research and improvements are necessary to ensure the long-term effectiveness and safety of Bt in agricultural production. Additionally, exploring integrated pest management strategies, combining biological, physical, and chemical control methods, is essential to achieve effective pest control while minimizing the impact on the environment and ecosystems.

## 4 Selection of Bt

In practical applications, biopesticides derived from Bt exhibit limitations, including a short persistent period and poor insecticidal efficacy. Hence, the future direction aims to enhance the toxicity and stability of Bt as an insecticide. Currently, numerous scholars have explored strategies and methods for optimizing the insecticidal activity of Bt. These include isolating and screening high-potency strains, genetically enhancing insecticidal crystals, utilizing genetic engineering to improve insecticidal genes, and increasing mutation rates through mutagenic agents, among others [19,20,21,22]. Gebremariam et al. [23] isolated 31 strains of Bt from 70 soil samples. However, only 20 strains (64.5%) proved virulent against the *greater wax moth*, with a maximum mortality rate of 95%. In another study, Barkad et al. [24] cloned the *lon* gene into a multi-copy vector, incorporating its original promoter and transcriptional terminator, and expressed it in *B. thuringiensis serovar israelensis* ATCC 35646. The results indicated efficient transcription and translation of the recombinant *lon* gene, leading to an improvement in the yield of intracellular toxins in the strain. Furthermore, the LC_50_ value of the *cry1Ac-cry9Aa* toxin decreased to 0.725 ng/cm^2^, lower than that of the control group. Additionally, Zhu et al. [25] identified a high-yield mutant strain of Bt, named BtX023PN, derived from the wild Bt X023. This strain was mutated using atmospheric and room temperature plasma and nitrosoguanidine. The virulence of BtX023PN against *Plutella xylostella* and *Mythimna seperata* increased by 2.33 and 2.13 times, respectively. Quantitative real-time PCR (qRT-PCR) and SDS-PAGE analysis revealed a 61% increase in the production of *Cry1Ac* protein.

Although many insecticidal genes of Bt were sequenced, it is still necessary to study their toxicity mechanism and actual insecticidal effect. Based on the optimization of Bt, it is also necessary to investigate the protein expression level, stability of insecticidal effect, genetic stability, and production technology. At the genome level, based on high-throughput sequencing technology and sequence similarity alignment technology, all potential insecticidal protein gene profiles of newly discovered strains were quickly predicted and analyzed at the DNA level; at the proteomics level, high-throughput and high-resolution tandem mass spectrometry can identify all potential insecticidal proteins produced by the strain at the whole proteome level. With the rapid progress of genome and proteome technology and data analysis, the analysis and identification of the complete spectrum of ICP genes in Bt have become the most convenient way. Based on the many advanced and efficient methods mentioned above, it is believed that Bt can overcome its obstacles and better benefit the humanity.

## 5 Research on Bt insecticides

### 5.1 Insecticidal toxin

The classification of Bt crystal toxins reveals the existence of 73 families and 6 groups of endotoxin proteins [26]. Each strain produces distinct types of toxins, which are either proteins or small molecules released outside the cell or stored within it. The toxins primarily include *δ*-endotoxins, exotoxins, hemolysins, enterotoxins, and vegetative insecticidal proteins (Vip’s). Notably, *δ*-endotoxins varying in molecular weight serve as the primary toxins for pest control. Also known as parasporal crystals, these toxins encompass the *Cry* protein and the *Cyt* protein. When ingested by larvae of Lepidoptera, Diptera, and Coleoptera, *δ*-endotoxins activate and decompose into small units of approximately 60 kDa, forming pores on the cell membrane, disrupting the osmotic pressure balance, leading to the lysis of midgut epithelial cells, and ultimately causing insect death [22]. Furthermore, some studies suggest that certain endotoxins eliminate pests by disrupting the function of intestinal cell mitochondria [27]. The *Cry* protein, with a molecular weight ranging from 72 to 134 kDa, is encoded by a single gene designated as “*Cry*.” This gene is further categorized into *CryӀ*, *CryⅡ*, *CryⅢ*, and *CryⅣ*. Specifically, the *CryӀ* gene exhibits insecticidal effects against Lepidoptera insects, while *CryⅡ* targets both Lepidoptera and Diptera. On the other hand, *CryⅢ* and *CryⅣ* are specific to Coleoptera and Diptera, respectively. Additionally, the *Cyt* protein demonstrates insecticidal activity against Diptera insects [28,29,30].

**Figure 3:**
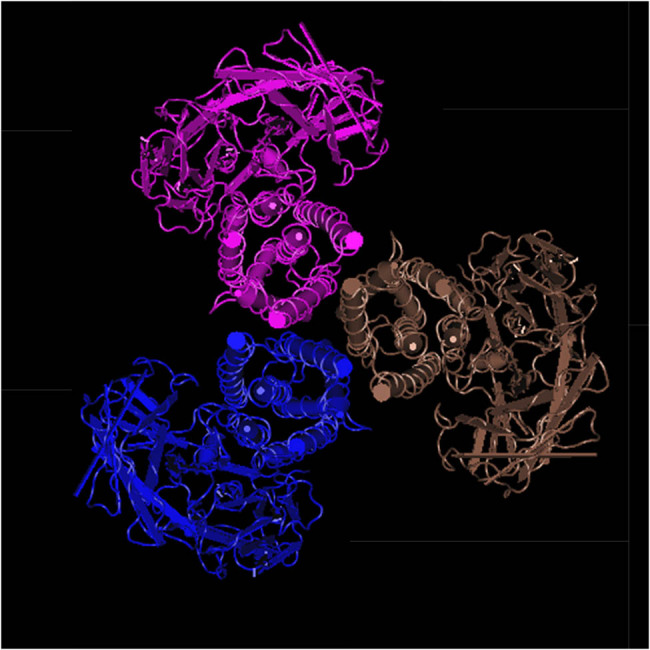
Three-dimensional structure of *Cry1B*.867.

The research on the structure and function of *δ*-endotoxins has now been relatively in-depth. The three-dimensional structure of the substance is shown in Figure 3, with three structural domains. Notably, domains I and II are intricately linked to its specific insecticidal activity [31]. Further investigations have revealed that domain II is the region where the protein primarily functions. Through mutagenesis experiments targeting several crucial residues within this region, it was discovered that these residues significantly impact the larvicidal activity of *δ*-endotoxin [32]. It can be seen that this area is very important.

### 5.2 Classification of ICPs

In 1998, Crickmore et al. [33] proposed a novel classification system for ICPs. This system employed an evolutionary tree algorithm to categorize and name the proteins into four distinct levels. Proteins with amino acid sequence homology below 45% were designated as the first level and represented by Arabic numerals. Proteins exhibiting homology between 45 and 78% fell into the second level and were represented by capital letters. Proteins with homology ranging from 78 to 95% were classified as the third level and designated with lowercase English letters. Finally, proteins with homology exceeding 95% comprised the fourth level and were represented by Arabic numerals. This comprehensive classification framework provides a useful tool for understanding and comparing the ICPs, facilitating further research and applications in biological pest control.

## 6 Application of Bt in agriculture and forestry

Bt overcomes the shortcomings of chemical pesticides, such as low safety, challenges in properly disposing of residues, significant environmental pollution, and the tendency to cause abnormal coloration in vegetables and fruits. Currently, Bt has emerged as the microbial insecticide with the largest production both domestically and internationally. It represents the most commercially successful and widely utilized microbial insecticide for controlling agricultural and forestry pests [34].

Bt is the largest and most widely used live microbial pesticide in China, and it is also an indispensable biological insecticide in many green and organic food production processes, accounting for over 10% of the total microbial pesticide market share [35]. These Bt biological insecticides are extensively utilized in the management of agricultural pests, forest and fruit tree pests, storage pests, as well as medical pests due to their outstanding economic, social, and ecological benefits. At present, the annual production of China’s Bt industry is about 30,000 tons (16,000 IU/µl), and the sales revenue in 2017 was about 100 million yuan [36]. This is sufficient to demonstrate the important role of Bt biopesticides in China’s agriculture and environmental protection work.

### 6.1 Bt formulation is directly used for pest control

Mazigo et al. [37] conducted a study in the Kilangali village, South Central Tanzania, where they utilized high-specific toxin-producing bacteria and Bt as biological larvicides in rice farmers. The findings revealed that over half (56.6%) of the participants reported a notable decrease in mosquito density within their households, while a quarter (26.6%) observed a reduction in the mosquito population on their farms. Notably, 93.3% of the participants reported that these measures effectively reduced the risk of malaria within their families. Eski et al. [38] carried out the insecticidal performance of 21 strains of *Bacillus*, especially *Agelastica alni* (Coleoptera: Chrysoptera), in order to develop an effective biopesticide. It was found that the *Bacillus thuringiensis* var.*tenebrionis*-xd3 (*Btt*-Xd3) was the best. To optimize the toxin protein and spore production of Xd3, the study determined the optimal growth conditions: a nutrient broth medium with salt, a pH of 7, and a temperature of 30°C. Under laboratory conditions, the lethal concentration 50 (LC_50_) of *Btt*-Xd3 against the target larvae was found to be 1.5 × 10^4^ c.f.u./ml. Consequently, a new biological pesticide, *Btt*-xd3, was developed from *B. thuringiensis* (Xd3), holding promise as a biological control agent against Coleoptera pests. Furthermore, Liu et al. [39] isolated a Bt strain, X023 (Btx023), from the Hunan Province, China, exhibiting high insecticidal activity. The study explored the effect of adding metals (Cu, Fe, Mg, and Mn) to the growth medium on the formation of spores and ICPs. Bioassays revealed that the wild strain Btx023 demonstrated significant insecticidal activity against *P. xylostella*. Notably, the addition of 1 × 10^−5^ M Cu^2+^ significantly enhanced the expression of *cry1Ac* and *vip3Aa* genes, leading to an increase in insecticidal activity. qRT-PCR and proteomic analysis further revealed that the upregulated proteins were involved in amino acid synthesis, the glyoxylate pathway, oxidative phosphorylation, and polyphosphorylation, among others. Copper ions appeared to play a crucial role in regulating these processes, which provided abundant raw materials for ICP synthesis.

### 6.2 Research on Bt genetically modified crops

The Bt gene has emerged as the most widely utilized transgenic insecticidal gene, attributed to its product, the Bt toxin protein, which boasts remarkable insecticidal effects, safety, and efficiency. Common Bt genetically modified crops, including tomatoes, corn, and cotton, have revolutionized agriculture. Canada pioneered in the commercialization of Bt genetically modified corn in 1996, which has since become widely cultivated in North America, significantly mitigating losses caused by corn pests [40]. China followed suit the next year, promoting this genetically modified corn to its farmers [41]. Since then, Bt genetically modified crops have gained global acceptance, leading to the development of numerous insect-resistant plant varieties. Statistics reveal that over 200 million ha of arable land worldwide are now planted with Bt genetically modified crops [42].

Coleoptera insects, renowned for causing severe damage to crops, pose a significant threat to crop yield and quality, thus making them a critical target for pest control efforts [43]. By the end of 2018, researchers had isolated and cloned a total of 850 Bt genes globally. Among these genes, *Cry1Ba*, *Cry3*, *Cry7*, *Cry8*, *Cry18*, *Cry23*, *Cry34*, *Cry37*, and *Cry43* have demonstrated a remarkable impact on most Coleoptera insects [44].

Pests, diseases, and weeds collectively cause significant losses to global food production. Statistics indicate that pests are responsible for 14% of annual food production losses, while diseases account for 10% and weeds contribute 11%. The economic toll of these harmful organisms is staggering, amounting to an annual loss of 120 billion yuan worldwide [45]. These organisms have become a critical biological factor hindering agricultural production. The application of Bt offers a promising solution, effectively reducing crop and forestry diseases and pests, ultimately benefiting the humanity.

## 7 Application of Bt in anticancer drugs

Cancer, a severe threat to human life, remains a challenging research area globally [46]. In recent years, certain bacterial proteins have garnered significant attention for their antitumor properties, such as *Pseudomonas aeruginosa* azurin [47] and *Corynebacterium diphtheriae* toxin [48], among these, proteins derived from non-insecticidal and non-hemolytic strains of Bt have demonstrated killing effects on various human cancer cells [49]. These proteins, specifically cytotoxic to human cancer cells, are known as “parasporins.” Parasporins (PSs), a class of bacterial proteins, can be categorized into six types: PS1, PS2, PS3, PS4, PS5, and PS6 [50]. Each type recognizes and eliminates cancer cells through distinct mechanisms. For instance, PS1 elevates Ca2^+^ levels and triggers apoptosis in multiple cancer cell types. PS2 functions as a cytolysin, targeting the plasma membrane of specific cancer cells. On the other hand, PS3 and PS6 act as pore-forming toxins, dissolving the plasma membrane of cancer cells. PS4 induces cancer cell death through a non-apoptotic pathway. The diverse mechanisms employed by PSs suggest that they may target different molecules and activate various signaling pathways in cancer cells [51]. The discovery of new PSs from Bt strains with specific cytotoxicity to human cancer cells has garnered increasing attention. For example, PS2 from Bt, a member of aerolysin-type β pore-forming toxins, has emerged as a potential therapeutic agent for cancer treatment due to its strong and selective cytotoxicity against human cancer cells without affecting normal cells [52]. For another example, Moazamian et al. [50] conducted a study in Iran, during which they isolated a total of 88 Bt strains. When the non-hemolytic crystal proteins of these isolates were treated with proteinase K, five strains belonging to three different biotypes of *B. thuringiensis –* namely, *thuringiensis*, *kurstaki*, and *sotto* – were identified to exhibit distinct cytotoxic effects against HCT-116 colon cancer cells and CCRF-CEM blood cancer cells. This finding suggests that the isolated Bt strains possess specific and different cytotoxicity toward human colon cancer and blood cancer cells.

The discovery of these strains with specific cytotoxicity is significant as it opens up new possibilities for the development of targeted anticancer therapeutics. The distinct cytotoxic mechanisms employed by these *B. thuringiensis* strains may offer novel approaches for the treatment of cancer. Future research on these strains could lead to the identification of novel bioactive components and the elucidation of their underlying mechanisms of action, potentially leading to the development of more effective and targeted anticancer drugs.

## 8 Function and application of Bt in environmental pollution control

### 8.1 Advantages of Bt in bioremediation

Bioremediation, a controlled or spontaneous process involving microorganisms, particularly stands out as a reliable environmental protection technology for water treatment. Its simplicity in operation, low investment requirements, minimal environmental interference, and absence of secondary pollution make it an increasingly popular choice for water environment treatment [53]. Among the various microorganisms capable of bioremediation, Bt has garnered significant attention [54]. Numerous studies have demonstrated the ability of Bt strains to effectively remove organic pollutants from both water and soil environments. These pollutants include, but are not limited to, petroleum hydrocarbons, pentachlorophenol, hexavalent chromium-contaminated wastewater, and organophosphorus pesticides [56,57,58]. Compared to other bacteria, Bt offers several distinct advantages. First, it is environmentally friendly and poses high safety standards, making it a suitable candidate for bioremediation applications. Second, the cost of using Bt is relatively low, which is a crucial factor in large-scale industrial applications. Finally, Bt is easy to cultivate and scale up for industrial fermentation, ensuring a consistent supply for bioremediation efforts. Despite its promising potential, there have been relatively few reports on the use of Bt for pollutant removal. This may be due to a lack of awareness or insufficient research in this area. However, given the established benefits of Bt in bioremediation and the increasing demand for sustainable and cost-effective water treatment solutions, it is anticipated that more research will be conducted in this direction in the future.

### 8.2 Pollutants degraded by Bt

Muñoz-Martínez et al. [59] isolated a strain of Bt from soil collected at a composting plant located northeast of Mexico City. This bacterium demonstrated remarkable potential in degrading bendiocarb, a commonly used insecticide. The study was conducted in a biofilm reactor, where the Bt strain was immobilized using fragments of volcanic rock, known as tezontle, as a support material. Operated on a continuous basis, this system achieved degradation rates exceeding 90% for the insecticide, displaying high removal efficiency. The utilization of Bt in this manner offers several advantages. First, the bacterium’s natural ability to degrade organic pollutants, including insecticides, makes it an ideal candidate for bioremediation applications. Second, the use of tezontle as a support material not only provides a stable environment for bacterial growth but also enhances the bioremediation process by increasing the surface area available for microbial attachment and degradation. This study demonstrates the potential of Bt in bioremediation, particularly in the degradation of insecticides. Its effectiveness in removing bendiocarb from water sources, combined with the ease of operation and low cost of the biofilm reactor system, makes it a promising technology for practical applications in water environment treatment. Future research could focus on optimizing the reactor conditions, exploring the degradation mechanisms of Bt, and extending its application to other types of pollutants.

In order to explore the effects and potential mechanisms of heavy metal-tolerant bacteria and biochar (BC) on reducing heavy metal accumulation in vegetables, Li et al. [60] tested Bt HC-2. The ability of BC and BC + HC-2 to fix Cd and Pb in culture medium was studied, and their effects on the dry weight and Cd of radish in metal-contaminated soil under field conditions were also investigated. The impact of Pb absorption and its potential mechanisms was also studied. These treatments significantly increased the dry weight of radish roots (18.4–22.8%) and leaves (37.8–39.9%) and decreased Cd (28–94%) and Pb (22–63%) contents in the radish roots compared with that of the control. Treatment with HC-2, BC, and BC + HC-2 also significantly increased the pH, organic matter content, NH4^+^ content, and NH4^+^/NO3^−^ ratio of rhizosphere soils and decreased the DTPA-extractable Cd (37–58%) and Pb (26–42%) contents in rhizosphere soils of radish. Furthermore, BC + HC-2 had higher ability than the other two treatments to protect radish against Cd and Pb toxicity and increased the radish biomass. Therefore, Bt HC-2 combined with BC can ensure vegetable safety *in situ* for the bioremediation of heavy metal-polluted farmlands.

## 9 Research on the excavation method of toxin proteins from Bt

Since the discovery in the early twentieth century that Bt can effectively kill pests, it has become an important issue to explore and utilize the efficient ICP of Bt in agricultural production and forestry pest control. From the early isolation of bacterial strains, followed by spraying pesticides rich in insecticidal proteins, to using molecular biology techniques to transform plants into expressing specific insecticidal proteins, humans have been continuously improving the accuracy of using ICPs in Bt. Therefore, continuously exploring new and usable genetic resources of Bt ICPs is a very important topic.

The rapid development of molecular biology technology in the 1980s and 1990s provided a technical foundation for the expression of Bt insecticidal proteins in target crops. The Bt toxin protein gene was first expressed in cotton in 1990 [61] and then successfully constructed and expressed in potatoes in 1995 [62]. The cloning and identification of insecticidal protein genes have become an important aspect of the development of Bt resources.

With the advent of high-throughput sequencing technology, the cost and time required for microbial genome sequencing have been significantly reduced. This remarkable progress has facilitated the exponential growth of genomic data, leading to a more comprehensive understanding of various microorganisms, including *Bacillus thuringiensis*. As of April 11, 2024, the NCBI website alone has published whole-genome sequencing data for 27 strains of Bt. These genomes have been annotated multiple times, resulting in 88 genome assembly and annotation reports, as well as 584 plasmid annotation reports (http://www.ncbi.nlm.nih.gov/gene). This makes it possible to high-throughput explore Bt toxin protein genes based on genomic data and mass spectrometry technology.

At the genomic level, high-throughput sequencing technology, coupled with sequence similarity alignment techniques, enables rapid prediction and analysis of all potential insecticidal protein gene maps of newly discovered Bt strains. This approach allows researchers to identify regions of the genome encoding insecticidal proteins, thereby facilitating the discovery of novel toxins or toxin variants; at the proteomic level, high-throughput and high-resolution tandem mass spectrometry techniques can identify all potential insecticidal proteins produced by strains at the entire proteomic level. The rapid development of genome and proteome technology, combined with data analysis capabilities, is the most effective method for the full spectrum analysis and identification of ICP genes in Bt. This approach holds promise for the discovery of new insecticidal proteins, which could potentially lead to the development of more effective and environmentally friendly pest control strategies.

### 9.1 Exploration and identification methods of insecticidal proteins based on DNA libraries

In the 1980s, genetic technology and molecular biology underwent rapid advancements, leading to a shift in the way researchers explored Bt insecticidal proteins. At that time, *Escherichia coli* was predominantly utilized to establish DNA libraries of these insecticidal proteins [63]. Subsequently, toxicological experiments were conducted on various pests to assess their insecticidal effects.

The core approach for discovering the insecticidal potential of Bt involved cloning the insecticidal protein gene onto suitable vectors [64]. This cloning process allowed researchers to determine the gene sequence encoding the insecticidal protein through gene sequencing technology. In 1983, Wong’s research group made a significant breakthrough by cloning and sequencing the gene regulatory sequence of ICPs, along with the DNA sequence encoding 333 amino acids at the N-terminus, which accounts for one fourth of the total length of ICPs [65].

Rabha and his colleagues [66] sequenced the entire genome of the *Bacillus thuringiensis* BA04 strain, isolated from soil samples in the Kashiranga National Park in Assam, Northeast India. This extensive sequencing effort aimed to understand the gene composition and identify genes responsible for producing insecticidal proteins, including virulence factors. They achieved great results: a total of 6,111 genes were discovered, including two new crystal protein coding genes (MH753632.1 and MH75363.1). These genes hold significant potential for developing insect-resistant genetically modified crops, thus contributing to sustainable agriculture. Additionally, this strain could be harnessed for the production of effective biopesticides, offering a natural and environmentally friendly approach to pest management.

### 9.2 Discovery methods based on a high-throughput genome sequencing technology

In recent years, the advent of high-throughput sequencing technology has revolutionized the exploration of *Cry* genes. This powerful tool enables the large-scale parallel sequencing of millions of DNA molecules, generating vast amounts of data at a reduced cost. This rapid and comprehensive analysis of the transcriptome and genome of Bt offers unprecedented insights, enabling researchers to solve problems that once took years to tackle.

Currently, researchers have developed a sophisticated high-throughput system designed to identify novel crystal protein genes (*Cry*) in Bt strains [67]. This innovative approach incorporates three distinct prediction methods: BLAST, Hidden Markov model, and support vector machine. These methods work in tandem to accurately predict *Cry* toxin genes within the genome. The performance of the system has been validated, demonstrating its remarkable speed, sensitivity, and specificity. With an average speed of 1.02 Mb/min for protein and open reading frames and 1.80 Mb/min for nucleotide sequences, it processes data swiftly, enabling the rapid analysis of large genomes.

With the decreasing cost of high-throughput genome sequencing technology, the publication of genomic data for Bt strains in public databases is growing exponentially. As the field of genomics continues to expand, researchers are poised to unlock even more secrets of Bt, leading to the development of more effective and sustainable biopesticides.

## 10 Synergism of insecticidal activity of Bt

Although Bt-based biopesticides are currently among the most effective and widely used biopesticides, their insecticidal activity and stability in practical applications often require enhancement through the use of synergists. These synergists, which are commonly employed to boost the performance of biopesticides, include a range of additives such as chemical additives, chemical insecticides, biological insecticides, and other enhancers [68].

The research conducted by Konecka et al. [69] has revealed a significant synergistic effect between the *Cry* toxin and carvacrol. When the bacterial toxin comprised 0.1 or 0.05% of the mixture, the combination was most effective in reducing the number of first instar and third instar *Spodoptera exigua* pests. The utilization of these mixtures led to a remarkable increase in insect mortality, exceeding the expected mortality by approximately 1.9 times. Furthermore, Akhanaev et al. [68] evaluated the effectiveness of a commercially available insecticide, Lepidocide, which is based on Bt var. *kurstaki* and *Lymantria dispar* multiple nucleopolyhedrovirus (LdMNPV). The study also investigated the enhanced efficacy of these components when combined with an optical brightener for controlling *L. dispar* larvae. The results demonstrated that most combinations of Lepidocide and LdMNPV, containing 5 mg/mL optical brightener, exhibited synergistic effects. These mixtures were highly effective in reducing the number of second instar larvae. Additionally, Soares Figueiredo et al. [70] were interested in understanding how combinatorial proteins interact with pests and whether combinatorial proteins contribute to resistance control and management. This work demonstrated the toxicity of Cry1Ab, Cry1Ac, Cry1Ca, Cry1Ea, Cry2Aa, Cry2Ab, Vip3Aa, and Vip3Ca in single and combined assays against *S. frugiperda* neonatal larvae. All protein mixtures had synergistic action in the control of the larvae. The Vip3Aa + Cry1Ab mixture had the highest toxicity, in the following sequential order: Vip3Aa + Cry2Ab, Cry1Ab + Cry2Ab + Vip3Aa, Cry1Ea + Cry1Ca, Cry1Ab + Cry2Ab, Vip3Ca + Cry1Ea, and Vip3Ca + Cry1Ca. Cry1Ab, Cry1Ac, Cry2Ab, and Vip3Aa were bound to more than one site on the brush border membrane vesicles of *S. frugiperda*. The Cry1Ab and Cry1Ac proteins share their binding sites, while Cry1Ab does not share its binding site with those of Cry2Aa and Cry2Ab proteins. The Vip3Aa protein does not share receptors with the tested *Cry1* and *Cry2*. The results suggest that a combination of these tested proteins may increase the toxicity against *S. frugiperda* neonates.

The selection of appropriate synergists is crucial to ensure compatibility with the biopesticide and to minimize any potential negative impacts on the environment or non-target organisms. Researchers are continuously exploring new synergistic options and optimizing their use to enhance the performance of Bt biopesticides, making them even more effective and sustainable tools for pest management.

## 11 Existing problems and prospects

Bt preparation, as a new microbial pesticide, is environment-friendly, has no effect to soil microorganisms, and can maintain the ecosystem balance. Moreover, its protein toxin is harmless to humans and other animals, has high safety, and is in line with the values of sustainable development and the inevitable trend of pesticide development in the future. However, there are still many problems to be solved for the large-scale application of Bt preparation.

(1) The resistance of pests can be inherited stably.
(2) Short insecticidal spectrum: in general, a strain can only be effective to its specific sensitive insects, and a single preparation cannot control multiple pests at the same time, so the insecticidal activity has limitations.
(3) Poor quick response, short duration, and unstable control effect: compared with traditional chemical pesticides, Bt preparation needs more time to exert its activity.
(4) The insecticidal effect is easily affected by the weather and environment: Bt preparation itself is a microbial insecticide, and the growth, reproduction, and activity of microorganisms are affected by many factors. For example, the most suitable growth temperature of Bt is 24–32°C, so its effect is poor when the temperature is lower than 15°C; the residual period of the preparation will be greatly reduced by the scouring of rainwater; meanwhile, the sporocystic insecticidal protein is easily inactivated after ultraviolet irradiation, etc.

In view of the above problems, a promising optimization direction is to select new high-efficiency Bt strains, reduce the cost of Bt fermentation, and improve the level of Bt fermentation. According to the mechanism of Bt preparation, it has become a research hotspot to reasonably unearth high-efficiency, stable, and environment-friendly synergistic substances, develop complex Bt preparation, and make up for the original defects. In addition, by constructing new strains through genetic engineering technology, we can obtain transgenic strains with a wider insecticidal spectrum and a high level of expression of ICPs.

On the basis of these studies, we can combine transcriptomics, proteomics, and metabonomics in the future to explore Bt more comprehensively. We believe that the development of quick acting, long-lasting, and broad-spectrum microbial agents will gradually reduce the use of or even completely replace the chemical pesticides in the near future.

## 12 Conclusions

To summarize, this article provides a comprehensive overview of the recent research achievements regarding the use of Bt in pest control. The key topics include its distinct features, the underlying mechanisms of action, application techniques, and synergism. Despite being the most extensively studied and applied biological insecticide, there are still challenges that urgently need to be addressed.

With technological advancements, Bt remains a prominent research focus in the realm of pest control, promising broader application prospects in the future. However, the current understanding of the molecular mechanisms and principles related to Bt, particularly its long-term safety for human health and the ecological environment, remains elusive. Therefore, further elucidating the molecular mechanisms and principles associated with Bt, as well as enhancing its safety evaluation system, is crucial not only for safeguarding the sustainable development of this biological insecticide but also for promoting the establishment of novel pest control strategies and fostering green agricultural practices.
